# Association of *Acinetobacter baumannii* EF-Tu with Cell Surface, Outer Membrane Vesicles, and Fibronectin

**DOI:** 10.1100/2012/128705

**Published:** 2012-05-15

**Authors:** Shatha F. Dallo, Bailin Zhang, James Denno, Soonbae Hong, Anyu Tsai, Williams Haskins, Jing Yong Ye, Tao Weitao

**Affiliations:** ^1^Department of Biology, The University of Texas at San Antonio, One UTSA Circle, San Antonio, TX 78249, USA; ^2^Department of Biomedical Engineering, The University of Texas at San Antonio, One UTSA Circle, San Antonio, TX 78249, USA; ^3^Department of Biology, The University of Texas at Austin, 1 University Station, Austin, TX 78712, USA; ^4^Pediatric Biochemistry Laboratory, The University of Texas at San Antonio, San Antonio, TX 78249, USA; ^5^Department of Chemistry, The University of Texas at San Antonio, San Antonio, TX 78249, USA; ^6^RCMI Proteomics and Protein Biomar Feers Cores, The University of Texas at San Antonio, San Antonio, TX 78249, USA; ^7^Center for Research & Training in The Sciences, The University of Texas at San Antonio, San Antonio, TX 78249, USA; ^8^Division of Hematology/Oncology, Department of Medicine, Cancer Therapy & Research Center, The University of Texas Health Science Center at San Antonio, San Antonio, TX 78229, USA; ^9^Department of Biology, College of Science and Mathematics, Southwest Baptist University, 1600 University Avenue, Bolivar, MO 65613, USA

## Abstract

A conundrum has long lingered over association of cytosol elongation factor Tu (EF-Tu) with bacterial surface. Here we investigated it with *Acinetobacter baumannii*, an emerging opportunistic pathogen associated with a wide spectrum of infectious diseases. The gene for *A. baumannii* EF-Tu was sequenced, and recombinant EF-Tu was purified for antibody development. EF-Tu on the bacterial surface and the outer membrane vesicles (OMVs) was revealed by immune electron microscopy, and its presence in the outer membrane (OM) and the OMV subproteomes was verified by Western blotting with the EF-Tu antibodies and confirmed by proteomic analyses. EF-Tu in the OM and the OMV subproteomes bound to fibronectin as detected by Western blot and confirmed by a label-free real-time optical sensor. The sensor that originates from photonic crystal structure in a total-Internal-reflection (PC-TIR) configuration was functionalized with fibronectin for characterizing EF-Tu binding. Altogether, with a novel combination of immunological, proteomical, and biophysical assays, these results suggest association of *A. baumannii* EF-Tu with the bacterial cell surface, OMVs, and fibronectin.

## 1. Introduction

A Gram-negative and obligate aerobic bacterial species, *Acinetobacter baumannii,* has emerged as one of the most important nosocomial pathogens [1–4], raising risks not only regional but also global in the aftermath of war and natural disasters.* A. baumannii* was identified in the US military personnel deployed to Iraq and Afghanistan [5]. Interestingly, more than 60% of the isolates were related to three pan-European clones that, in fact, had been disseminated in geographically distinct areas [6]. Besides, *Acinetobacter *infections are associated with natural disasters, such as the 1999 earthquake in Turkey [7] and the 2008 earthquake in China [8].

For such austerity of *A. baumannii *infections, little is known about the pathogenesis. To explore the fundamental mechanisms, we tested whether extracellular proteins of *A. baumannii *mediate the bacterial attachment [9]. Proteins were extracted from whole-cell lysate, outer membrane (OM) fractions, and cell-free spent cultures (CFCs) of the wild-type and the biofilm mutants of *A. baumannii *we isolated [9]. With a proteomic approach, translation elongation factor (EF-Tu) of *A. baumannii* was detected in cell-free cultures, the data suggesting release of EF-Tu from the bacterial cells. The release appeared unlikely to result from cell death and lysis but rather likely to be regulated, because the mutants, as viable as the wild type, exhibited deficiency in the release and cell adhesion [9]. The EF-Tu release seemed to be a puzzle to us as the primary function of EF-Tu, while remaining to be characterized for *A. baumannii*, is translation elongation as deduced from the *E. coli* EF-Tu, because EF-Tu and translation are highly conserved throughout the bacterial domain [10–12]. Specifically, in the first step of peptide chain elongation on ribosomes, EF-Tu*·*GTP serves as a carrier of codon-specified aminoacyl-tRNA to the ribosomal aminoacyl site. Eubacterial EF-Tus belong to the superfamily of GTP-binding proteins. It is not a membrane protein, since EF-Tu lacks a signal sequence and transmembrane domains that mediate protein translocation across cell membrane.

This has led to a conundrum concerning EF-Tu release. The original clue to this question may come from a study with the sucrose-dependent spectinomycin-resistant mutants of *Escherichia coli* grown in the absence of sucrose [13]. EF-Tu was detected in the OM fractions; its presence in OM did not result from artificial binding during membrane preparation. It was also found in the periplasm of *Neisseria gonorrhoeae *[14]. Two decades after the initial finding, *E. coli* EF-Tu was detected again in the OM fractions of the cells adherent to abiotic surface [15]. The bacterial surface association of EF-Tu has been further evidenced by EF-Tu involvement in *Staphylococcus aureus* biofilm development [16], in mediating attachment to human cells by *Lactobacillus johnsonii *[17] or *P. aeruginosa *[18]. The EF-Tu surface association has been attested by its acting as a part of pathogen-associated molecular patterns recognized by receptors on eukaryote hosts [19], as a target for a serine-threonine phosphatase involved in virulence and survival of* Listeria monocytogenes *in the infected host [20], and as an active protein eliciting innate [21] and acquired immunity [16, 22].

How the surface-associated EF-Tu is released still seems to be an enigma. Our hypothesis was that *A. baumannii *EF-Tu is associated with outer membrane vesicles (OMVs). The rationale is based on the proteomic analyses that have implicated EF-Tu association with OMVs in multiple bacterial species [23] and with OM in *A. baumannii* [24], and *A. baumannii* actually produces OMVs [25]. To test it, we cloned and sequenced the EF-Tu encoding gene, purified the recombinant EF-Tu (rEF-Tu), and produced EF-Tu antibodies. Then we employed a combination of transmission electron microcopy (TEM), proteomics, Western blot, and an optical sensor to show that EF-Tu is associated with OMVs and OM and binds to the host extracellular matrix protein fibronectin.

## 2. Results

### 2.1. A. baumannii EF-Tu

**Figure 1:**
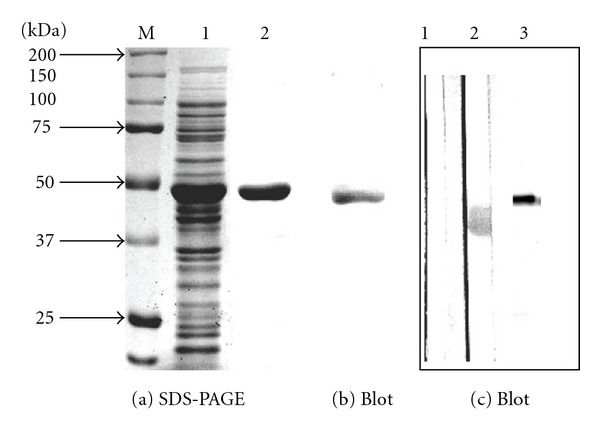
Purification of *A. baumannii* EF-Tu. Purification of *A. baumannii* rEF-Tu. (a) Overexpressed (lane 1) and column-purified rEF-Tu (lane 2). (b) Immunoblot of column-purified rEF-Tu with anti-His-tag monoclonal antibody. (c) Immunoblot of *A. baumannii* cell lysate with rabbit prebleed (lane 1) and anti-rEF-Tu antibodies (2). Immunoblot of rEF-Tu with the anti-rEF-Tu (3).

The EF-Tu encoding gene of **A. baumannii ** ATCC19606 strain was sequenced and the protein was purified for antibody development. The ATCC 19606 strain was chosen for novelty because its genome was not completely sequenced and the EF-Tu encoding gene was not studied at the time we started our investigation. The availability of genome sequencing data for the ATCC 17978 strain greatly facilitated our study. Based on the genome data, there are two genes for EF-Tu, namely *tufAa* and *tufBa,* both identical [26], with reference to *tufAe *and* tufBe *of* E. coli.* The *tufAe* deletion caused *E. coli* growth defect in rich media, while the *tufBe* deletion did not [27], the observations suggesting that *tufAe* is functional. These data led us to clone and sequence *tufAa *of the* A. baumannii* 19606 strain. Comparison of the *tufAa* sequences from 17978 and 19606 strains showed 99.8% identity; the small difference resulted from two nucleotide changes located in 1,032 and 1,137 (Figure S1 in Supplementary Material available online at doi: 10.1100/2012/128705)—GCA of the 19606 strain but GCG of the 17978 strain—a silent mutation in the codon for alanine. The gene of the 19606 strain was cloned and His-tagged; rEF-Tu (48 kDa) was expressed and purified to homogeneity (Figure 1(a) lane 2). Immunoblots of the His-tagged rEF-Tu showed that the tagged rEF-Tu reacted with anti-His monoclonal antibodies (b), verifying that the purified protein was His-tagged. The identity of rEF-Tu was confirmed with proteomic analysis as we described before [9]. Furthermore, the antiserum specific to rEF-Tu was produced. Immunoblots with the sera indicate that the antiserum recognized both 43 kDa EF-Tu in cell lysate (Figure 1(c) lane 2) and 48 kDa rEF-Tu in the purified fraction (lane 3), but the preimmune serum did not (lane 1). The band of EF-Tu from the whole-cell extract appeared wider (lane 2) than that from the purified fraction (lane 3), suggesting that EF-Tu undergoes slight degradation in the cell extract, in line with the previous data about cleavage of *E. coli* EF-Tu by a phage-exclusion system [28].

### 2.2. EF-Tu Associated with OMVs and Cell Surface of A. baumannii

**Figure 2:**
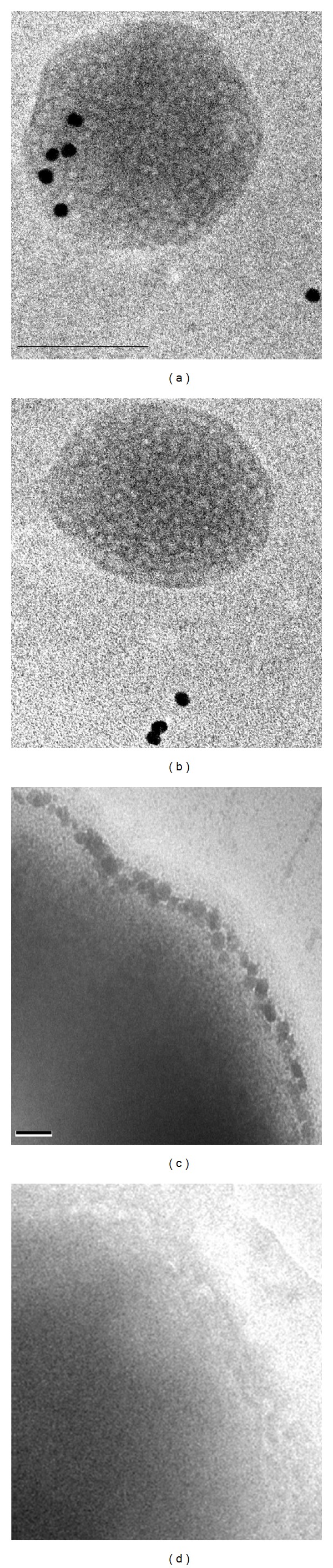
. EF-Tu visualized on *A. baumannii *OMVs and cells by immune TEM with EF-Tu antibodies. Immunogold TEM with anti-EF-Tu antibodies showing localization of (a) EF-Tu on the isolated OMV and (c) on cell surface of *A. baumannii*. Immunogold with preimmune serum shows no localization on OMVs (b) and cells (d). *n* = 40 OMVs. Bar: 100 nm.

**Figure 3:**
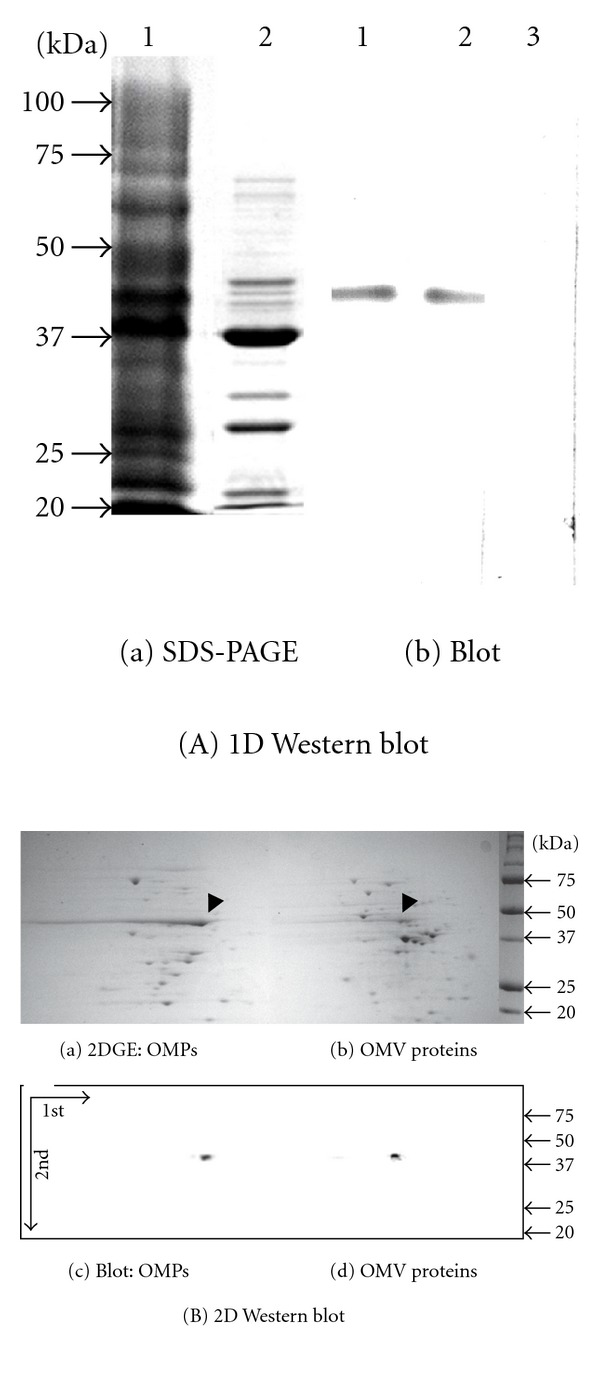
EF-Tu detected in OMV and OM fractions. Panel (A): (a) SDS-PAGE of proteins from OM (lane 1) and OMV fractions (2). (b) Western blot of proteins from OM (lane 1) and OMV fractions (2) reacted with the EF-Tu antibody diluted at 1 : 3000. Control blot with pre-immune serum (lane 3). Panel (B): 2D-based Western blot. Proteins from OM (a and c) and OMV fractions (b and d) were resolved by isoelectric focusing (1st D) and then separated on a second dimension SDS-PAGE (2nd D). Proteins from the gel were blotted onto PVDF membranes and probed with the anti-EF-Tu antibodies (c and d). Arrows: EF-Tu. Blot with pre-immune serum not shown.

Immune TEM of **A. baumannii ** OMVs and the cells was conducted with the antibodies specific for rEF-Tu in order to examine whether EF-Tu is physically associated with *A. baumannii *OMVs and cell surface. OMVs or cells were incubated with the anti-rEF-Tu antibodies or the pre-immune serum as a control. After washes, the samples were probed with the gold-labeled anti-IgG antibodies and examined under TEM (Figure 2). When OMVs were probed with the primary EF-Tu antibodies and the secondary gold-labeled antibodies, high-density dots of gold particles were often observed associated with OMVs (a). When the cells were probed, the gold particles appeared to deposit on the cell surface (c), the result consistent with a previous finding with thin sectioning of bacterial cells [17]. In contrast, when the pre-immune serum was used, the gold dots were mostly washed off (b, d). The rEF-Tu antibodies appeared highly specific not only for rEF-Tu (Figure 1(c) lane 3) but also for EF-Tu in the cell lysate (Figure 1(c) lane 2), the OM, and the OMV fractions (Figure 3). Evidently, deposition of the gold-labeled antibodies specific for the EF-Tu antibodies on OMVs and cells appeared reflective of EF-Tu on the surfaces of OMVs and the cells. The results provide the physical evidence for association of EF-Tu with OMVs and cell surface of *A. baumannii*.

### 2.3. EF-Tu Detected in OM and OMV Fractions

The physical association of EF-Tu with OMVs and cell surface prompted us to verify the presence of EF-Tu in the OM and the OMV subproteomes. We performed 1D and 2D gel-based Western blotting analyses. First, we resolved the proteins of the OM (lane 1) and the OMV fractions (lane 2) in SDS-PAGE (Figure 3(A)(a)). After the proteins in the gel were transferred onto a membrane and probed with the anti-rEF-Tu antibodies (Figure 3(A)(b)), a protein band of 43 kDa the same as the EF-Tu mass was detected in the OM (lanes 1) and the OMV fractions (lane 2) [10]. In contrast, when the membrane was probed with the pre-immune serum, this protein band was not detected (lane 3). Second, to address the limitation of 1D resolution, we conducted the 2D gel electrophoresis and probed the proteins for EF-Tu (Figure 3(B)). EF-Tu was still detected by the antibodies in the OM (a, c) and the OMV fractions (b, d), but not by the pre-immune serum control.

While the evidence of Western blot for presence of EF-Tu in the OM and the OMV fractions appeared to be convincing, there were some drawbacks of Western blotting analyses, such as lack of scope due to limitation on utilizing costly arrays of antibodies to target multiple proteins. Proteomic analyses of the OMV and the OM subproteomes were carried out to address them. Briefly, total proteins in the lane of SDS-PAGE as shown in Figure 3(A)(a) were subjected to the *in vitro* trypsin proteolysis and capillary LC/MS/MS. The degraded peptide masses were determined and searched across the bacterial protein databases with the *P* < 0.05-based MOWSE scoring algorithm [29]. By this significance threshold and the cut-off score of 50, 144 proteins were identified in the OMV and the OM fractions (Table 1). EF-Tu was detected in both OMV and OM subproteomes (no. 57 in Table S2). The consistent results of EF-Tu obtained by the immunological and the proteomic analyses attested the validity of both methodologies in detection of EF-Tu. The proteomic analyses also detected OmpA (no. 51) in the OM and the OMV fractions, the results consistent with the former finding [25]. The consistent results of the immune TEM with the Western blotting and proteomic analyses demonstrate that EF-Tu is indeed associated with OMVs, unlikely due to protein contamination during protein sample preparation.

### 2.4. The OMV- or OM-Associated EF-Tu Binding to Fibronectin

**Figure 4:**
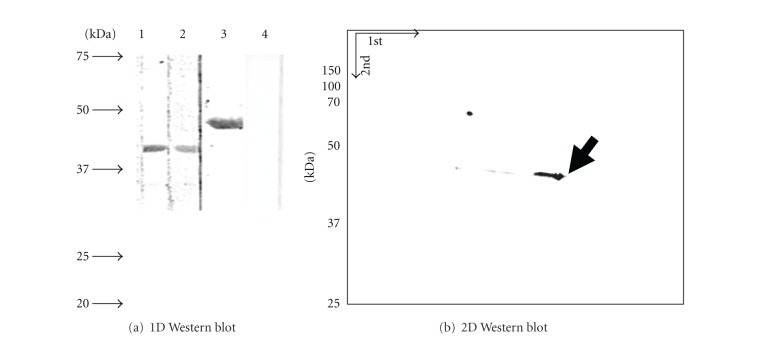
Binding of EF-Tu to fibronectin examined by Western-blot-based binding assays. (a) Proteins were resolved by SDS-PAGE and blotted onto PVDF membranes. Proteins from OM (lane 1) and OMVs (2) and the purified rEF-Tu (3) were probed with FN and anti-FN. rEF-Tu (4) and proteins from OM (not shown) probed with anti-FN alone. (b) Immunoblot of 2-DE of proteins from OM probed with FN followed by anti-FN. Arrow indicates EF-Tu confirmed by protein sequencing.

As *A. baumannii* EF-Tu was detected in OMVs and on the bacterial cell surface (Figures 2 and 3) and *Mycoplasma pneumoniae* EF-Tu was found to bind the host extracellular matrix protein fibronectin [30], it could be hypothesized that the OMV- and the OM-associated EF-Tus of *A. baumannii* bind to fibronectin. This hypothesis was tested with the Western-based binding assays. The proteins extracted from the OM (lane 1 of Figure 4(a)) and the OMV fractions (lane 2) were fractioned together with rEF-Tu (lane 3) by SDS-PAGE and transferred onto PVDF membranes. The membrane strips (1, 2, and 3) were incubated with fibronectin and then blotted against the fibronectin antibodies. A band of 43 kDa corresponding to the EF-Tu mass was detected in the OM (lane 1) and OMV fractions (lane 2). A 48 kDa band known to be rEF-Tu (lane 3) was detected but not by the pre-immune serum (lane 4). Identity of EF-Tu in each of the bands was confirmed by proteomic analysis as we described previously [9]. To deal with the limited power of 1D resolution, we performed the 2-D gel electrophoresis of the proteins from OM (Figure 4(b)) and probed the proteins for fibronectin binding as above. One conspicuous spot was seen, having a size of 43 kDa; the protein identity was determined to be EF-Tu by proteomic analysis as above. Evidently, the fibronectin-EF-Tu complexes were recognized by the fibronectin antibodies.

**Figure 5:**
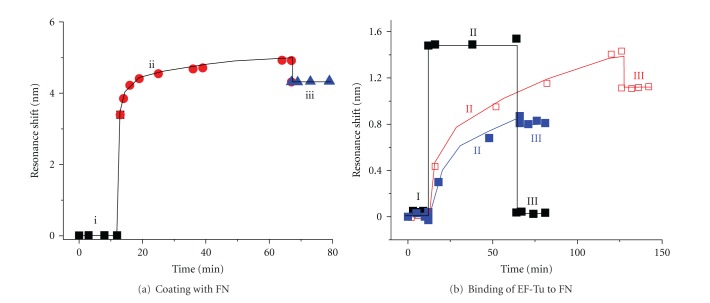
Interaction of EF-Tu and fibronectin characterized by a PC-TIR sensor. Binding of an analyte to a reactant causes resonant wavelength shift that was measured throughout the process as a function of time. (a) Coating the sensor with fibronectin. (i) The sensor surface was calibrated with PBS and (ii) coated with fibronectin (FN at 200 *μ*g/mL in a 200 *μ*L volume of PBS at 25°C). Immobilization of FN onto the sensor caused resonant wavelength shift. (iii) The subsequent washes. (b) Binding of EF-Tu to FN. (I) Detection baseline of the FN functionalized sensor. (II) EF-Tu at 20 *μ*g/mL (blue curve) or 50 *μ*g/mL (red) and a negative control (a portion of proteoglycan 4) at 20 *μ*g/mL (black) were incubated with FN at 25°C for the indicated times. (III) The sensor was washed.

Binding of rEF-Tu to fibronectin was further characterized by a novel label-free optical sensor (Figure 5). The sensor is based on a photonic-crystal structure in a total-internal-reflection (PC-TIR) configuration. The unique working principle and high sensitivity of the PC-TIR sensor have been demonstrated by Ye and his colleagues [31–34]. The assays with the PC-TIR sensor encompass two steps: sensor coating and protein binding, each including three phases. For coating as indicated in Figure 5(a), (i) the baseline was calibrated with PBS; (ii) the sensor was coated with fibronectin (200 *μ*g/mL) and the coating was detected by measuring the resonant wavelength shift; (iii) the subsequent washes removed the unbound protein molecules, leading to a minor reduction in the resonance shift, but the substantial changes still remained indicating the effective coating of the sensor by fibronectin. The binding thickness of fibronectin on the sensor was determined to be 4.8 nm corresponding to the 4.2 nm resonance shift calculated with a transfer matrix method [31]. For protein binding as demonstrated in Figure 5(b) (blue), after (I) the coating baseline was calibrated, (II) EF-Tu was added onto the fibronectin-coated sensor surface. A gradual increase in resonance shift was observed, demonstrating EF-Tu binding to fibronectin. (III) The unbound or weakly bound protein molecules were washed off subsequently. The resonance shift level remained high after wash, indicating strong binding of EF-Tu to fibronectin. When the EF-Tu concentration increased from 20 to 50 *μ*g/mL, the resonant shift level changed from 0.8 nm to 1.1 nm (blue and red in Figure 5(b)). The binding thicknesses of EF-Tu are 0.91 nm and 1.26 nm, respectively. The reason that the binding thickness did not increase as much as the increase of the protein concentrations may be attributed to binding saturations. In a sharp contrast, after addition of the proteoglycan 4 control, resonant shift was not observed; the resonant wavelength returned to the baseline after wash (Black).

## 3. Discussion

A combination of biological, immunological, biophysical, and proteomic methods was employed to test the hypothesis stating that EF-Tu is associated with OMVs and binds to fibronectin. The results revealed by immune TEM show that EF-Tu was physically associated with the OMVs and the cell surface. Its presence in the OMV and the OM subproteomes was verified by Western blotting and proteomic analyses. EF-Tu carried by OMVs and OM was found to bind to fibronectin as detected with the Western blot- and the PC-TIR-based sensor assays.

### 3.1. OMV-OM Subproteomes and Possible Mechanisms for EF-Tu Delivery into OMVs

**Figure 6:**
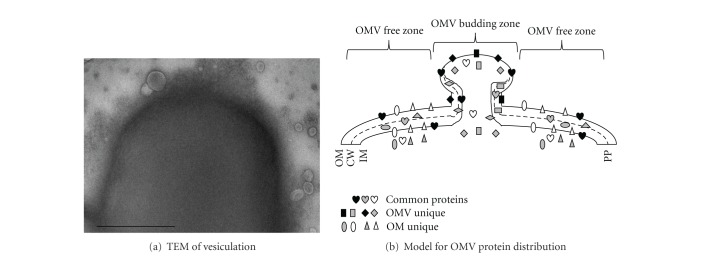
EF-Tu delivery model. (a) A cell with budding and released OMVs revealed by TEM. Bar: 250 nm. (b) A model derived from the OM and the OMV subproteomic data as well as the immune TEM observations, depicting an OMV budding upwards (see Discussion for details). OM: outer membrane; CW: cell wall; IM: inner membrane; PP: periplasm. Proteins in three subproteomes (common, OMV unique and OM unique) are indicated therein.

Our subproteomic analyses provide clues to the mechanisms of EF-Tu delivery into OMVs. We categorized the proteins in the OMV and the OM subproteomes (Table 1). The first consisted of the proteins detected only in the OMV subproteome (Table S1). The second was the common category of the proteins shared by the two subproteomes (Table S2). The third comprised the proteins present only in the OM subproteome (Table S3). While the biological meaning of these subproteomes remains to be deciphered, the current finding implies that there may be the OMV-budding zones from which OMVs bud from OM. The budding might be random as indicated in Figure 6(a). If so, OMVs produced via random budding should have displayed irregular protein distributions; yet this presumption does not appear reconciled with the subproteomic observations. Rather, our data let us suggest the OMV budding zones for protein delivery into OMVs (Figure 6(b)). First, the proteins of the OMV subproteome seem to be located in the OMV budding zones from which OMVs bud out and carry these proteins with OMVs (Table S1). Second, the common proteins seem likely to scatter over the two zones (Table S2). Third, the proteins of the OM subproteome seem to be distributed in the OMV-free zones and so not to be seen in OMVs (Table S3). Based on the premise, EF-Tu that belongs to the OM-OMV common subproteomes seems to scatter over the OMV-budding and the OMV-free zones; it seems to be delivered into OMVs through OMV budding from OM (Figure 6(b)). This model can be used to explain the presence of EF-Tu in the OM, the OMVs, and the CFC fractions. Understandable is the absence of the OM-only proteins (e.g., #78, 43 kDa glucose-sensitive porin; #91, 46 kDa urocanase; #96, putative aromatic compound porin; #109, 43 kDa l-sorbosone dehydrogenase in Table S3) from the OMVs (Table S2) and the CFC fractions. In *Bacillus subtilis, *EF-Tu localizes underneath the cell membrane, colocalizing and interacting with MreB, an actin-like cytoskeletal element that plays a role in cell shape maintenance [35]. These predictions may stimulate future studies for their verification.

### 3.2. EF-Tu and Other Cytosolic Proteins in OMV and OM Subproteomes

One of the intriguing observations is the presence of cytosolic proteins in the OMV and the OM subproteomes, for example, DNA binding proteins (#18, 26, 45) in OMV and EF-Tu (#57) in the common subproteome. Considering OM's hydrophobic nature, we were tempted to suspect cytosolic protein contamination. Nevertheless, detection of DNA-binding proteins in OMVs seems unlikely to be attributed to contamination, as DNA-binding proteins and DNA were detected with in *N. gonorrhoeae *OMVs [36, 37]. Since DNA was detected in *A. baumannii* OMVs (Figure S2), it seems possible that the proteins are hitched by DNA into OMVs or vice versa. Moreover, the presence of EF-Tu in both OMV and OM subproteomes is not just coincident but consistently documented [23]. Its presence in OM did not result from artificial binding during membrane preparation [13]. EF-Tu was detected in both subproteomes of multiple species [23]. EF-Tu was found in OM fractions of *A. baumannii* [24]. It also was present in OMVs of *N. meningitides *[38, 39] and *E. coli *[40]. However, a former proteomic analysis did not detect EF-Tu in the *A. baumannii* OMV fraction [25]. This discrepancy with ours may be due to different strains and growth conditions used in their and our studies. Kwon et al. used *A. baumannii *from clinical isolates, but we employed the standard ATCC19606 strain. They grew the culture under shaking condition while we used plates. While a combination of physical, immunological, and biochemical evidence appears to be convincing, we plan to compare data acquired from the standard and the clinical strains concerning the discrepancy.

### 3.3. Implications for Binding of *A. baumannii* EF-Tu to Fibronectin: *A. baumannii* EF-Tu Bound to Fibronectin (Figures 4 and 5)

 The role of the binding seems to be intriguing. EF-Tus of* L. johnsonii *[17] and *P. aeruginosa *[18] are involved in bacterial attachment to human cells. Particularly, EF-Tu is involved in bacterial infection of human monocyte-like cells via binding to the cell-surface-associated nucleolin [41]. Given that fibronectin was found to bind to macrophage as documented [42] and that *A. baumannii* EF-Tu was detected in OMVs and on the bacterial cell surface, these data seem to support a notion that *A. baumannii* EF-Tu contributes to mediating adhesion of the bacterial cells and OMVs to macrophages through binding to fibronectin on the host cells, a hypothesis to be tested in the future.

## 4. Experimental Procedures

### 4.1. Expression and Purification of EF-Tu

The gene *tufAa* encoding EF-Tu of *A. baumannii *was cloned by following the manufacturer instruction (Novagen, San Diego, CA, USA). Since the genome of the *A. baumannii* 19606 strain was not available when we conducted this study, *tufAa* of *A. baumannii* 17987 was used for designing specific primers and PCR was performed with the 19606 DNA template. Forward primer: 5′- GACGACGACAAGATGATGGCTAAAGCCAAG-3′ and the reverse primer: 5′-GAGGAGAAGCCCGGTCCGTCACTATATTATGCTTATGC-3′. The PCR product (1,191 bp) was cloned into the pTriEX-4 Ek/LIC expression vector (Novagen, San Diego, CA, USA), which added an N-terminal hexa-histidine (6xHis) for purification by affinity chromatography and S-tag. The positive recombinant EF-Tu (rEF-Tu) clone DNA was verified by DNA sequencing analysis. The expressed rEF-Tu was fused with N-terminus His-S-tags containing 48 amino acids and purified by nickel affinity chromatography under native conditions by following the manufacturer protocol (Qiagen). The purified protein was analyzed with SDS-PAGE to determine the presence of rEF-Tu and was confirmed further by immunoblot by using monoclonal antibody to His-tag. The identity of rEF-Tu was further confirmed by N-terminal microsequencing as we described before [9].

### 4.2. Preparation of Antibody against rEF-Tu

The antiserum specific to rEF-Tu was developed by ProSci Incorporated. Briefly, rabbits were injected subcutaneously with 100–200 *μ*g rEF-Tu with complete Freund's adjuvant. Individual rabbits were boosted 3 times with the same amount of antigen in incomplete Freund's adjuvant at intervals of 21 days. Serum samples were collected and used in immunological studies. ProSci Incorporated provided us with sera prior to immunization and 3 bleed collected serums.

### 4.3. Isolation of OMV and OMPs

*A. baumannii *(ATCC19606) OMVs were isolated from LB agar plates as described previously [43] with modifications. Colonies grown on LB agar plates were scraped off and suspended in PBS with gentle agitation to OD_600 nm_ of 5. Then, bacteria were collected by low-speed centrifugation (6,000 g) for 5 min, and the recovered supernatant was centrifuged at 12,000 g for 10 min and further passed through the 0.2-*μ*m pore size filters (Millipore). OMVs in the supernatant were then collected by ultra centrifugation at 100,000 g for 12 hours at 4°C and resuspended in PBS. OMPs were extracted according to Caldwell et al. [44].

### 4.4. TEM and Immunogold TEM

TEM was conducted according to a standard protocol [25, 45]. For immunogold TEM, *A. baumannii* cells and purified OMVs were immunogold-labeled with the anti-EF-Tu antibodies as described previously with some modifications [30]. Briefly, the cells and OMVs were incubated with 100 mM Tris-HCl buffer (pH 7.5) containing 1% bovine serum albumin (BSA) supplemented with 1% heat inactivated goat serum (buffer A) to reduce nonspecific binding. They were incubated with anti-EF-Tu diluted 1 : 100 in buffer A at 37°C for 2 hrs, washed with buffer A, and incubated with goat anti-rabbit immunoglobulin-G- (IgG-) gold complex (average size particle, 10 nm, 1 : 20 dilution) suspended in PBS containing 1% BSA (buffer B). After sequential washing with buffer B, PBS, and deionized water, bacterial cells and OMVs were mounted onto Holey carbon film nickel grids by fixing with 1% glutaraldehyde-4% formaldehyde for 20 min at room temperature. Grids were stained with 7% uranyl acetate followed by Reynolds lead citrate for TEM.

### 4.5. Western Blot

 Proteins from OMV and OM fractions were separated on 10% SDS-PAGE [46] and stained by Coomassie blue. The proteins were transferred electrophoretically onto nitrocellulose membranes [47]. The membrane strips were incubated with the *A. baumannii* anti-rEF-Tu antibodies at a dilution of 1 : 3000 in 1% (w/v) blotto for 2 hrs at 25°C. The membrane strips then were washed, incubated in alkaline phosphatase-conjugated goat anti-rabbit antibodies (Santa Cruz Biotechnologies, Santa Cruz, CA), at a dilution of 1 : 5000 in 1% (w/v) blotto for 1 hr at 25°C, washed, and developed with 5-bromo-4-chloro-3-indolyl phosphate/Nitroblue Tetrazolium (BCIP/NBT, Sigma). For 2D gel electrophoresis, proteins from OMV and OM fractions were solubilized for isoelectric focusing (IEF) in 8 M urea, 2% CHAPS, 2% IPG buffer (Amersham Biosciences), 20 mM DTT, and traces of bromophenol blue. Sample was loaded on a 13 cm Immobiline DryStrip pH 3–10 (Amersham Biosciences) by rehydration and left under oil overnight. IEF was conducted under 17,000 Vh by using the Multiphor II (Amersham Biosciences) at 20°C. The IEF strips were equilibrated in Equilibration solution (0.05 M Tris-HCl, 0.4 M urea, 30% glycerol, 1% SDS (w/v), and 0.02 M DTT) for 10 minutes. SDS-PAGE (10% w/v) was conducted as previously [46]. After separation of the supernatant proteins by electrophoresis, the proteins in the gels were transferred electrophoretically onto nitrocellulose membranes [47]. Membranes were blocked and incubated with the anti-rEF-Tu at a dilution of 1 : 5000 in 1% blotto for 1 hr, washed, and developed with Sigma FAST BCIP/NBT solution.

### 4.6. OM-OMV Subproteomic Analyses

 The analysis was performed as described previously [45, 48]. Briefly, OMPs were isolated as above, and OMV proteins were extracted by resuspending in 2% (w/v) SDS and 100 mmol l^−1^ DTT and incubating at 25°C for 5 min. The proteins (30 *μ*g) were fractionated by SDS-PAGE (10%, w/v). After staining, the proteins-containing lane in replicate was sliced into pieces (1 × 1 mm) for *in vitro* trypsin proteolysis. Capillary liquid chromatography-tandem mass spectrometry (LC/MS/MS) was performed, and the peptides derived from the proteins in the gel slices were determined with a linear ion trap tandem mass spectrometer in which the top 7 eluting ions were fragmented by collision-induced dissociation. Proteins were identified by following a standard protocol [49], in which MS/MS spectra were searched against the NCBI nonredundant protein database (version 20100306; 10551781 sequences and 3596151245 residues) with a probability-based database searching algorithm (Mascot, Matrix Science). A score of each peptide entry was calculated by the molecular weight search (MOWSE) peptide-mass database developed previously [29] and the scoring algorithm. The significance threshold was set for *P* ≤ 0.05 in a search for random matches, and the proteins consistently detected in the replicates were counted.

### 4.7. Binding of Released EF-Tu to Fibronectin

 Nitrocellulose membranes transferred with proteins from OMV and OM fractions were blocked, washed, and incubated with pure fibronectin (Sigma, human plasma fibronectin) at 10 *μ*g/mL for 24 hr at 4°C. Then, the membranes were washed and incubated with the rabbit antifibronectin antibodies at 1 : 10,000 dilution in 1% blotto for 2 hr at room temperature. Subsequently, the membranes were washed and incubated with the alkaline-phosphatase-conjugated goat anti-rabbit antibodies at a dilution of 1 : 20,000 in 1% blotto for 1 hr, washed, and developed as above.

### 4.8. Functionalization of PC-TIR Sensor

The sensor originates from a prototype of a photonic crystal structure in a total-internal-reflection (PC-TIR) configuration [33]. The design and fabrication of the PC-TIR sensor was reported by Ye and his coworkers [31, 32, 34]. Briefly, a photonic crystal (PC) structure with five alternating layers of silica and titania was fabricated on a transparent BK7 glass substrate by electron beam physical vapor deposition. A thin film of poly(methyl methacrylate) (PMMA) (A6, MicroChem) was spin-coated on the top of the structure at 500 rpm for 10 sec, followed by 4,200 rpm for 45 sec. The sensor chip was baked at 60°C for 30 minutes. Two sample wells were fabricated with polydimethylsiloxane and placed in a tight contact with the top surface of the sensor chip. One is used as the reference channel and the other as the signal channel. Both wells were filled with PBS, and the resonant wavelengths of the two channels were recorded to establish the detection baseline. Fibronectin was immobilized on the chip surface [50] through physical absorption by directly adding fibronectin (200 *μ*L, 200 *μ*g/mL) on the sensor chip followed by incubation at 25°C for the indicated time as in Figure 5. Shift in resonant wavelength was measured and recorded. The sensor surface was washed with PBS twice and refilled with 200 *μ*L PBS for measuring the thickness of bound protein. Finally, PBS was replaced with the analyte solution of EF-Tu or the proteoglycan 4 control in PBS (100 nM, 200 *μ*L). For the preparation of the control, the DNA (1.2 kb) encoding a portion of proteoglycan 4 was cloned; the polypeptide (44 kDa) was expressed and purified from baboon temporomandibular joint cells; it was tested negative in fibronectin binding by the same antibody-based assay as shown in Figure 5 (unpublished data by Jennifer McDaniel Schulze et al.). 

## Supplementary Material

The Supplementary Materials include sequences of the *A. baumannii* gene for EF-Tu (Fig. S1), electrophoresis analysis of *A. baumannii* OMV DNA (Fig. S2), as well as proteins present only in OMV (Table S1) and in OM subproteomes (Table S3), and proteins shared by OMV and OM subproteomes (Table S2).

## Figures and Tables

**Table 1:** Summary of OMV and OM subproteomes.

Subproteomes	SP*-TM** domains (*n*)	TM (*n*)	None (*n*)	Total (*n*)
OMV	12% (6)	59% (29)	29% (14)	34% (49)
OM	46% (32)	29% (20)	25% (17)	48% (69)
Common	39% (10)	27% (7)	34% (9)	18% (26)
	33% (48)	39% (56)	28% (40)	100% (144)

*Signal peptide; **transmembrane domains. Common: proteins detected in both OMV and OM fractions.
