# Supplementary material for: Association of Acinetobacter baumannii EF-Tu with Cell Surface, Outer Membrane Vesicles, and Fibronectin
Source: ScientificWorldJournal. 2012 May 15;2012:128705. doi: 10.1100/2012/128705 (PMC3362023; doi:10.1100/2012/128705)
Supplement: Supplementary file 1 — The Supplementary Materials include sequences of the A. baumannii gene for EF-Tu (Fig. S1), electrophoresis analysis of A. baumannii OMV DNA (Fig. S2), as well as proteins present only in OMV (Table S1) and in OM subproteomes (Table S3), and proteins shared by OMV and OM subproteomes (Table S2). [file 128705.f1.pdf]

Supplementary information

ATG GCT AAA GCC AAG TTT GAA CGT AAT AAA CCA CAC GTA AAC GTG GGT ACA ATC GGT
CAC GTT GAC CAT GGT AAA ACA ACT TTA ACT GCT GCG ATT GCA ACA ATT TGT GCA AAA ACT
TAC GGC GGT GAA GCG AAA GAT TAC TCA CAA ATC GAC TCA GCA CCT GAA GAA AAA GCA CGT
GGT ATT ACA ATT AAT ACA TCA CAC GTA GAA TAC GAT TCT CCA ACT CGT CAC TAC GCA CAC
GTT GAC TGC CCA GGC CAC GCC GAC TAC GTT AAA AAC ATG ATC ACT GGT GCT GCT CAG ATG
GAC GGC GCG ATC CTT GTA TGT GCT GCA ACT GAC GGT CCA ATG CCA CAA ACT CGT GAG CAC
ATC CTT CTT TCT CGT CAG GTA GGT GTA CCT TAC ATC ATC GTA TTC TTA AAC AAA TGC GAC
CTT GTT GAT GAC GAA GAA TTA CTT GAA TTA GTA GAA ATG GAA GTA CGT GAA CTT CTT TCT
ACT TAT GAC TTC CCA GGT GAT GAC ACT CCA GTA ATC CGT GGT TCA GCT CTT GCA GCG CTT
AAC GGT GAA GCT GGT CCT TAC GGT GAA GAA TCA GTT CTT GCT CTT GTA GCA GCA CTT GAC
TCT TAC ATC CCA GAG CCA GAG CGT GCA ATC GAC AAA GCA TTC TTG ATG CCA ATC GAA GAC
GTA TTC TCA ATT TCT GGT CGT GGT ACA GTA GTA ACA GGC CGT GTT GAA GCT GGT ATC ATC
AAA GTT GGT GAA GAA GTA GAG ATC GTT GGT ATT AAA GAT ACA GTT AAA ACA ACT GTA ACT
GGC GTA GAA ATG TTC CGT AAA CTT CTT GAC GAA GGC CGT GCA GGT GAG AAC TGT GGT ATC
TTA CTT CGT GGT ACT AAG CGT GAA GAA GTA CAA CGT GGT CAA GTA CTT GCT AAA CCA GGT
ACA ATC AAG CCG CAC ACT AAA TTC GAC GCA GAA GTA TAC GTA CTT TCT AAA GAA GAA GGT
GGT CGT CAC ACT CCA TTC TTA AAT GGT TAC CGT CCA CAG TTC TAC TTC CGT ACA ACT GAC
GTA ACT GGT GC**A** ATC CAG TTG AAA GAA GGC GTT GAA ATG GTA ATG CCA GGT GAC AAC GTT
GAA ATG TCA GTA GAA TTA ATC CAC CCA ATC GCA ATG GAC CCA GGT CTA CGT TTT GC**A** ATC
CGT GAA GGT GGT CGT ACT GTA GGT GCT GGT GTT GTT GCT AAA GTA ACT GCA TAA

Fig. S1. Sequences of the *A. baumannii* gene for EF-Tu. *tufAa* of the *A. baumannii* 19606 strain was cloned and sequenced. Its sequences were compared with those of the 17978 strain, and 99.8% homology was found. The changes in two nucleotides were located in 1,032 and 1,137, highlighted by shadow and boldness.

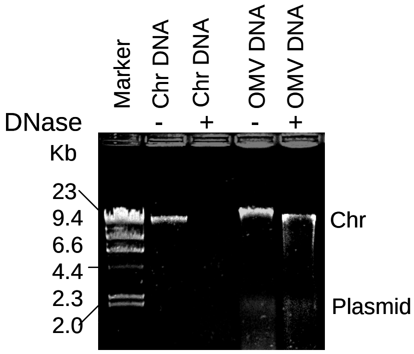

Fig. S2. Electrophoresis analysis of *A. baumannii* OMV DNA. OMV DNA was examined by agarose gel electrophoresis. OMV DNA consisted of chromosomal (chr) DNA (~23 kb) and small-sized DNA (~2 kb). The short DNA is likely to be plasmid, a prediction based on a previous report (15). DNase (0.005 U per µg DNA) was used to cleave extracellular DNA. Although the OMV chr DNA appeared partially cleaved, the OMV plasmid, albeit small and faint, was not fully degraded. One possible interpretation is that the plasmid DNA is enclosed in OMVs that protect the DNA from DNase sufficiently as reported previously (15), while the chrDNA is associated with OMVs outwardly that provide partial protection as described before (10). DNA in OMVs is aligned with DNA-binding proteins detected in OMVs.

Table S1 Proteins present only in OMV subproteome

	Proteins in OMV fraction	Score*	Mass	Domains SP[‡], TM[§]	Predicted functions	Database ID
1.	Succinyl-CoA synthetase subunit alpha [*A. baumannii* SDF]	272	30674	n,TM	TCA cycle	gi|169632628
2.	Hypothetical protein A1S_2710 [*A. baumannii* ATCC 17978]	223	42234	n,TM	TCA	gi|126642741
3.	Malate dehydrogenase [*A. baumannii* ATCC 17978]	148	32400	n,TM	Glycolysis	gi|126643040
4.	CsuE [*A. baumannii*]	139	36554	SP,TM	Type I pili assembly	gi|37933551
5.	Branched-chain amino acid aminotransferase [*A. baumannii* ACICU]	131	34408	n,TM	Branched chain family amino acid metabolic process	gi|184159475
6.	Glutamate dehydrogenase [NAD(P)+] oxidoreductase protein [*A. baumannii* ATCC 17978]	131	40128	n,TM	Arginine biosynthesis	gi|126643149
7.	Dihydrolipoamide succinyltransferase, component of 2-oxoglutarate dehydrogenase complex (E2) [*Acinetobacter* sp. ADP1]	127	43454	n,TM	TCA	gi|50085915
8.	ketol-acid reductoisomerase [*A. baumannii* SDF]	122	36830	n,n	Amino-acid biosynthesis	gi|169634382
9.	Succinyl-CoA synthetase beta chain [*A. baumannii* ATCC 17978]	118	36757	n,TM	TCA	gi|126642749
10.	L-glutamine synthetase [*Novosphingobium aromaticivorans* DSM 12444]	108	51875	n,TM	Glutamine biosynthetic process	gi|87199130
11.	Acyl-CoA dehydrogenase A [*Acinetobacter* sp. M-1]	107	68279	n,TM	Oxidation reduction	gi|21717403
12.	Methylcitrate synthase [*A. baumannii* SDF]	106	43955	n,TM	TCA	gi|169632107
13.	Dihydrolipoamide S-acetyltransferase E2 component of the pyruvate dehydrogenase complex [*A. baumannii* ATCC 17978]	104	64889	n,TM	Glycolysis	gi|126643334
14.	Glutaminase-asparaginase [*A. baumannii* ATCC 17978]	101	31037	SP, TM	Asparagine metabolic process	gi|126641512
15.	Putative UTP-glucose-1-phosphate uridylyltransferase [*A. baumannii* ATCC 17978]	100	29032	n,n	Biosynthetic process	gi|126640175
16.	AdeK [*A. baumannii* ATCC 17978]	98	50128	SP, TM	Transporter	gi|126642768
17.	Polynucleotide phosphorylase [*A. baumannii* ATCC 17978]	98	71168	n,TM	mRNA catabolic process	gi|126640439
18.	Integration host factor subunit alpha [*A. baumannii* ATCC 17978]	97	10339	n,n	DNA recombination, transcription, translation regulation	gi|126640672
19.	Aspartate-semialdehyde dehydrogenase [*A. baumannii* ATCC 17978]	96	40542	n,TM	Amino-acid biosynthesis	gi|126640500
20.	Malic enzyme [*A. baumannii* SDF]	94	82742	n,TM	Oxidation reduction	gi|169632939
21.	Glyceraldehyde-3-phosphate dehydrogenase [*A. baumannii* ATCC 17978]	88	49788	n,n	Glycolysis	gi|126642537
22.	Hypothetical protein A1S_0097 [*A. baumannii* ATCC 17978]	85	13056	n,n	Unknown	gi|126640204
23.	Hypothetical protein ACICU_01297 [*A. baumannii* ACICU]	78	55249	n,TM	Unknown	gi|184157617
24.	Dihydrolipoamide dehydrogenase [*Psychrobacter arcticus* 273-4]	75	51110	n,TM	Redox homeostasis	gi|71064685
25.	Succinylarginine dihydrolase [*A. baumannii* ATCC 17978]	75	49834	n,n	Arginine metabolism	gi|162286718
26.	DNA-binding protein HU-beta [*A. baumannii* ATCC 17978]	68	9316	n,n	Bacterial histone-like protein family.	gi|126641682
27.	N-acetyl-gamma-glutamyl-phosphate reductase [*A. baumannii* ATCC 17978]	68	33392	n,TM	Arginine biosynthesis	gi|126641958
28.	Leucyl aminopeptidase [*A. baumannii* ATCC 17978]	67	45775	n,TM	proteolysis	gi|126640320

29.	Bifunctional succinylornithine transaminase [*Yersinia pseudotuberculosis* IP 32953]	66	44188	n,TM	Arginine metabolism	gi|51596285
30.	(3R)-hydroxymyristoyl-ACP dehydratase [*A. baumannii* ATCC 17978]	65	16335	n,TM	Lipid A biosynthesis	gi|126642011
31.	6,7-dimethyl-8-ribityllumazine synthase [*Acinetobacter* sp. ADP1]	65	16411	n,n	Riboflavin biosynthesis	gi|50086524
32.	Acetyl-CoA acyltransferase [*A. baumannii* SDF]	63	41062	n,TM	Lipid degradation	gi|169634579
33.	Biotin carboxyl carrier protein of acetyl-CoA carboxylase (BCCP) [*A. baumannii* ATCC 17978]	63	13235	n,TM	Fatty acid biosynthetic process	gi|126642055
34.	Putative UDP-glucose 4-epimerase [*A. baumannii* ATCC 17978]	62	31131	SP, TM	Galactose metabolic process	gi|126640178
35.	Aspartate carbamoyltransferase catalytic subunit [*Polaromonas* sp. JS666]	61	34659	n,TM	Pyrimidine biosynthesis	gi|91787042
36.	β-ketoacyl-ACP synthase 1 [*A. baumannii* ATCC 17978]	61	28728	n,TM	Biosynthetic process	gi|126640913
37.	Cysteine synthase A [*A. baumannii* SDF]	61	35111	n,TM	Cysteine biosynthesis	gi|169632185
38.	2-C-methyl-D-erythritol 2,4-cyclodiphosphate synthase [*Leifsonia xyli* subsp. xyli str. CTCB07]	60	39273	n,TM	Isoprene biosynthesis	gi|50955383
39.	Arsenate reductase [*Rhodopseudomonas palustris* HaA2]	59	16396	n,TM	Oxidation reduction	gi|86749094
40.	Hypothetical protein LJ0783 [*Lactobacillus johnsonii* NCC 533]	56	62619	n,TM	ATP biosynthetic process	gi|42518708
41.	Malate:quinone oxidoreductase [*A. baumannii* ATCC 17978]	55	60397	SP, TM	TCA	gi|162286745
42.	30S ribosomal protein S13 [*Acinetobacter* sp. ADP1]	54	13141	n,n	Translation	gi|50086193
43.	Hypothetical protein glr1304 [*Gloeobacter violaceus* PCC 7421]	54	96551	n,TM	TonB box	gi|37520873
44.	Hypothetical protein FP1533 [Flavobacterium psychrophilum JIP02/86]	53	84894	n,n	Unknown	gi|150025584
45.	TraI nickase/helicase [*Yersinia pestis*]	51	188825	n,n	Conjugation	gi|52788159
46.	L-asparaginase, type II [*Clostridium botulinum* A str. ATCC 3502]	51	36511	n,n	Amino acid metabolic process	gi|148380471
47.	CoA-substrate-specific enzyme activase domain-containing protein [*Campylobacter curvus* 525.92]	50	156226	n,n	Unknown	gi|154173735
48.	Hypothetical protein CPF_2364 [*Clostridium perfringens* ATCC 13124]	50	122950	SP, TM	Unknown	gi|110799698
49.	Predicted 3',5'-cyclic-nucleotide phosphodiesterase [uncultured bacterium BAC13K9BAC]	50	29015	n,n	cAMP phosphodiesterase activity	gi|68304930

*, Score is based on *P* that is ≤0.05; ‡, signal peptide; §, transmembrane domains.

Table S2 Proteins shared by OMV and OM subproteomes

	Common proteins in both OMV and OM fractions	Score* OM/OMV	Mass	Domain SP‡,TM§	Predicted function	Database ID
50.	Putative protein (DcaP-like) [*A. baumannii* ATCC 17978]	1889/522	44818	SP,TM	Unknown	gi|126642784
51.	OmpA [*A. baumannii* ATCC 17978]	1737/240	37342	SP,TM	Porin	gi|169632583
52.	CsuA/B [*A. baumannii* AYE]	750/1885	18694	SP,TM	Type I pili assembly	gi|50083922
53.	23 kDa OMP putative [*A. baumannii* ATCC 17978]	659/74	22472	SP,TM	OmpA family	gi|126640934

54.	GroEL [*A. baumannii* SDF]	443/490	56886	n,TM	Chaperone	gi|126642873
55.	48 kDa OMP putative [*A. baumannii* AYE]	349/116	47833	SP,TM	Porin activity	gi|160222634
56.	Major intrinsic multiple antibiotic resistance efflux outer membrane protein precursor [*Acinetobacter* sp. ADP1]	130/91	52747	SP,TM	Transporter activity	gi|169632592
57.	Elongation factor Tu [*A. baumannii* ATCC 17978]	100/53	42760	n,n	Protein biosynthesis	gi|184157280
58.	Putative signal peptide [*A. baumannii* ATCC 17978]	91/59	16557	SP,TM	Unknown	gi|169794337
59.	Glutamine synthetase [*A. baumannii* ACICU]	86/132	52166	n,TM	Glutamine biosynthetic process	gi|126641619
60.	30S ribosomal protein S7 [*Acinetobacter* sp. ADP1]	79/46	17614	n, n	Translation	gi|126642245
61.	Catechol 1,2-dioxygenase [A. *radioresistens*]	77/55	33527	n,n	Catechol catabolic process	gi|126642195
62.	Oxidoreductase [*A. baumannii* ATCC 17978]	77/39	23477	n,TM	Oxidoreductase activity	gi|50084093
63.	ATP synthase subunit alpha [*A. baumannii* ATCC 17978]	72/100	55363	n, TM	ATP synthesis coupled proton transport	gi|183598067
64.	ATP synthase subunit beta [*Acinetobacter* sp. ADP1]	71/108	50280	n,n	ATP synthesis coupled proton Transport	gi|16118478
65.	Putative RND type efflux pump (AdeT) [*Acinetobacter* sp. ADP1]	71/142	36705	SP,TM	Efflux pump involved in aminoglycoside resistance	gi|50083469
66.	Aspartate aminotransferase A [*A. baumannii* ATCC 17978]	66/117	40652	n, TM	Biosynthetic process, aminotransferase	gi|169795946
67.	Succinylglutamic semialdehyde dehydrogenase [*Burkholderia pseudomallei* K96243]	56/61	51806	n,n	Arginine catabolic process	gi|126642261
68.	RecF [*Halorhodospira halophila* SL1]	56/42	39466	n,n	DNA replication and repair	gi|126641943
69.	CsuB [*A. baumannii* ATCC 17978]	56/56	14678	SP,TM	Type I pili assembly	gi|50083793
70.	GlcD [uncultured marine bacterium]	51/35	33668	n,n	Oxidoreductase activity	gi|168699716
71.	AtpA [uncultured Geobacter sp.]	47/51	29023	n,TM	ATP synthesis coupled proton transport	gi|34558039
72.	50S ribosomal protein L2 [*Acinetobacter* sp. ADP1]	43/63	30294	n,n	Translation	gi|126642262
73.	CsuA [*A. baumannii* ATCC 17978]	43/64	16058	SP,TM	Type I pili assembly	gi|218961916
74.	Putative acetyl-CoA acetyltransferase [*A. baumannii* AYE]	41/99	40774	n,TM	Acetyl-CoA C-acetyltransferase activity	gi|150389285
75.	50S ribosomal protein L9 [*Acinetobacter* sp. ADP1]	40/89	15775	n,n	Translation	gi|94499932

*, Score is based on *P* that is ≤0.05; ‡, signal peptide; §, transmembrane domains.

Table S3 Proteins detected only in OM subproteome

	Proteins in OM fraction	Score*	Mass	Domains SP‡, TM§	Predicted functions	Database ID
76.	AdeC-like OMP [*A. baumannii* AYE]	658	52901	SP,TM	Transport, lipid binding	gi|126640934
77.	34 kDa OMP [*A. baumannii* ACICU]	599	32095	SP,TM	Cell OM	gi|50085972
78.	Glucose-sensitive porin (OprB-like), putative [*A. baumannii* ATCC 17978]	463	42919	SP,TM	Transporter	gi|126643304
79.	Hypothetical protein ABSDF0721 [*A. baumanni* SDF]	460	47029	SP,TM	Unknown	gi|126642784
80.	Hypothetical protein ABSDF2360 [*A. baumanni* SDF]	333	41181	SP,TM	Unknown	gi|169797605

81.	CsuD [*A. baumanni* ATCC 19606]	244	92613	SP,TM	Fimbrium biogenesis	gi|72535027
82.	OM receptor protein [*A. baumanni* ACICU]	238	82433	SP,TM	Fe transport	gi|260554509
83.	Elongation factor Ts [*A. baumanni* ATCC 17978]	212	30691	n,n	Protein biosynthesis	gi|72535025
84.	Hypothetical protein BACUNI_00110 [Bacteroides uniformis ATCC 8492]	204	66417	n,TM	Arabinose catabolism	gi|126642362
85.	OM receptor for monomeric catechols [*A. baumanni* ACICU]	202	81197	SP,TM	Siderophore-iron transmembrane transporter	gi|160887709
86.	ComL, putative [*A. baumannii* ATCC 17978]	189	40583	SP,TM	Competence protein	gi|184156805
87.	Long-chain fatty acid ABC transporter [*A. baumanni* ACICU]	178	53090	n,TM	Short-chain fatty acid transporter activity	gi|126640892
88.	Electron transfer flavoprotein alpha-subunit [*A. baumanni* SDF]	146	31492	n,TM	Electron carrier activity	gi|169794882
89.	Hypothetical protein ABSDF1556 [*A. baumanni* SDF]	141	35019	SP,TM	Unknown	gi|169632682
90.	50S ribosomal protein L4 [*Acinetobacter* sp. ADP1]	133	22084	n,n	Translation	gi|169633229
91.	Urocanase [*A. baumanni* ATCC 17978]	108	45525	n,TM	Histidine catabolic process	gi|50086214
92.	Hypothetical protein ABAYE2569 [*A. baumanni* AYE]	105	11220	SP,TM		gi|126643414
93.	Peptidoglycan-associated lipoprotein precursor [*A. baumanni* ATCC 17978]	105	18215	SP,TM	Cell wall	gi|169796592
94.	Hypothetical protein ACICU_00960 [*A. baumanni* ACICU]	102	53061	SP,TM	Unknown	gi|126642630
95.	Glutathione peroxidase [*A. baumanni* ATCC 17978]	97	20138	n,n	Glutathione peroxidase activity	gi|50083988
96.	MTA/SAH nucleosidase, putative [*Acinetobacter* sp. ADP1]	97	31412	SP,TM	Amino-acid biosynthesis	gi|126666815
97.	Putative aromatic compound porin [*A. baumanni* AYE]	95	47905	SP,TM	Porin activity	gi|126640260
98.	OMP W, putative [*A. baumanni* SDF]	93	23123	SP,TM	Unknown	gi|169795795
99.	Hypothetical protein ABAYE0137 [*A. baumanni* AYE]	92	28124	SP,TM	Unknown	gi|126642014
100.	Hypothetical protein A1S_3051 [*A. baumanni* ATCC 17978]	91	33668	SP,TM	Unknown	gi|126641100
101.	Electron transfer flavoprotein beta-subunit [*A. baumanni* ATCC 17978]	89	23050	n,n	Electron carrier activity	gi|126643237
102.	Two-component response regulator [*A. baumanni* ATCC 17978]	89	22493	n,n	Two-component signal transduction	gi|169634030
103.	Hypothetical protein A1S_1574 [*A. baumanni* ATCC 17978]	87	9353	SP,TM	Unknown	gi|126642663
104.	50S ribosomal protein L15 [*Acinetobacter* sp. ADP1]	85	15419	n,n	Translation	gi|184158870
105.	Alkyl hydroperoxide reductase, C22 subunit, thioredoxin-like (detoxification of hydroperoxides) [*Acinetobacter* sp. ADP1]	85	20715	n,n	Redox homeostasis	gi|50086196
106.	NADH-dependent enoyl-ACP reductase [*A. baumanni* ATCC 17978]	84	28626	n,TM	Fatty acid biosynthetic process	gi|50085223
107.	OmpH, putative [*A. baumanni* ATCC 17978]	81	17857	SP,TM	Protein binding	gi|149369942
108.	30S ribosomal protein S8 [*Acinetobacter* sp. ADP1]	80	14146	n,n	Translation	gi|126642013
109.	L-sorbosone dehydrogenase [*A. baumanni* ATCC 17978]	79	43072	SP,TM	Catalytic activity	gi|126640348
110.	Hypothetical protein A1S_1879 [*A. baumanni* ATCC 17978]	76	15550	SP,TM	Unknown	gi|10440998
111.	30S ribosomal protein S9 [*Acinetobacter* sp. ADP1]	75	14263	n,n	Translation	gi|126641924
112.	PapD [*A. baumanni* ACICU]	74	30596	SP,TM	P pilus assembly chaperone	gi|50086030
113.	AdeB RND protein [*Acinetobacter* baumannii]	72	112542	n,TM	Transporter activity	gi|162286757
114.	50S ribosomal protein L5 [*Coxiella burnetii* RSA 493]	68	20735	n,n	Translation	gi|50083305
115.	Siderophore receptor, putative [*A. baumanni* ATCC 17978]	67	55201	n,TM	Transporter activity	gi|29653602
116.	OM usher protein, putative [*A. baumanni* AYE]	66	94546	SP,TM	Transporter activity	gi|126642125
117.	Indole-3-glycerol-phosphate synthase [*A. baumanni* SDF]	65	29909	n,n	Aromatic amino acid biosynthesis	gi|126642544

118. Dienelactone hydrolase, putative [*A. baumanni* ATCC 17978]	64	19173	n,n	Hydrolase activity	gi|169632923
119. Hypothetical protein A1S_0505 [*A. baumanni* ATCC 17978]	64	36477	SP,TM	Unknown	gi|126643437
120. Serine/threonine transporter SstT [*A. baumanni* ATCC 17978]	63	32591	n,TM	Amino acid transport	gi|49617981
121. Hypothetical protein N9414_21524 [Nodularia spumigena CCY9414]	62	56954	n,n	Unknown	gi|126641583
122. Rod shape-determining protein [*Acinetobacter* sp. ADP1]	62	36755	n,TM	Cell morphogenesis	gi|119513654
123. Lipoprotein, putative [*A. baumanni* ATCC 17978]	61	24108	SP,TM	Lipoprotein	gi|50084039
124. Bifunctional aconitate hydratase 2/2-methylisocitrate dehydratase [Pseudomonas aeruginosa PAO1]	58	93569	n,TM	Lyase	gi|160935071
125. Hypothetical protein STM2315 [Salmonella typhimurium LT2]	58	64600	n,TM	Unknown	gi|73531089
126. Nucleoside diphosphate kinase (NDK) (NDP kinase) (nucleoside-2-P kinase) [*Acinetobacter* sp. ADP1]	57	15438	n,TM	Nucleotide metabolism	gi|161523490
127. Hypothetical protein CPn0336 [Chlamydophila pneumoniae CWL029]	56	35334	SP,TM	Thiamine biosynthesis	gi|118558
128. Hypothetical protein ROS217_02710 [Roseovarius sp. 217]	56	79867	n,TM	Methyltransferase activity	gi|126643008
129. Pantoate-beta-alanine ligase [Helicobacter hepaticus ATCC 51449]	56	31207	n, n	Pantothenate biosynthesis	gi|53719993
130. Phosphopyruvate hydratase [*A. baumanni* ATCC 17978]	56	42941	n,TM	Glycolysis, cell surface	gi|15618256
131. Quinoprotein glucose dehydrogenase A	56	86902	SP,TM	Oxidation reduction	gi|85707540
132. 3-oxoacyl-[acyl-carrier-protein] synthase III [*Pedobacter* sp. BAL39]	55	35569	n,TM	Lipid synthesis	gi|86559631
133. 50S ribosomal protein L10 [*A. baumanni* SDF]	55	18079	n,n	Translation	gi|149277213
134. Acyl-CoA synthetase [*Clostridium perfringens*]	55	56370	n,TM	Fatty acid catabolism	gi|121998015
135. Bifunctional aldehyde dehydrogenase/enoyl-CoA hydratase [*A. baumanni* ATCC 17978]	53	69794	n,TM	Oxidation reduction	gi|24375681
136. Major OMP 1 [*Chlamydia trachomatis*]	53	42867	SP,TM	Porin activity	gi|126641381
137. AraC family transcriptional regulator [*Pseudomonas mendocina ymp*]	52	20399	n,n	Transcription regulation	gi|16330496
138. Hypothetical protein ANACOL_00094 [*Anaerotruncus colihominis* DSM 17241]	52	48918	n,TM	Protein biosynthesis	gi|149924971
139. Acetyl-CoA hydrolase [Shewanella denitrificans OS217]	51	44835	n,TM	Acetyl-CoA metabolic process	gi|168703700
140. Bacitracin resistance protein BacA [*Gemmata obscuriglobus* UQM 2246]	51	29255		Bacitracin resistance	gi|167768777
141. Hypothetical protein ABAYE2389 [*A. baumannii* AYE]	51	15768	SP,TM	Serine-type endopeptidase inhibitor activity	gi|112981082
142. Nitrite reductase [*Gemmata obscuriglobus* UQM 2246]	51	87173		Nitrate usage	gi|169796428
143. Major facilitator transporter [*Enterobacter* sp. 638]	50	44786	n,TM	Multidrug efflux pumps	gi|126640887
144. Ribonuclease HII [*Chlamydophila pneumoniae* AR39]	50	33573	n,n	Endonuclease	gi|146311149

[*], Score is based on *P* that is ≤0.05; [‡], signal peptide; [§], transmembrane domains.
